# Effect of spatial arrangement of presynaptic calcium channels on the calcium current cooperativity of neurotransmitter release

**DOI:** 10.1186/1471-2202-12-S1-P298

**Published:** 2011-07-18

**Authors:** Victor Matveev, Richard Betram, Arthur Sherman

**Affiliations:** 1 Department of Mathematical Sciences, New Jersey Institute of Technology, Newark, NJ, USA; 2 Department of Mathematics, Florida State University, Tallahassee, FL, USA; 3 Laboratory of Biological Modeling, NIDDK, National Institutes of Health, Bethesda, MD, USA

## 

Interaction between calcium nanodomains of individual voltage-dependent calcium channels in triggering exocytosis in neurons and endocrine cells is of significant importance for understanding localized calcium signaling in general and synaptic physiology in particular. Such channel domain interaction (overlap) is often experimentally probed by measuring the sensitivity of release rate to the total presynaptic calcium current, which is varied by changing the number of open channels while the single-channel current is kept fixed. Variation in the number of activated channels is in turn achieved using appropriate voltage-clamp protocols, or by pharmacologically blocking a subset of channels. The resulting slope of the log-log regression line between release rate and total calcium current is termed calcium current cooperativity, and is believed to indirectly probe the number of channels contributing to the exocytosis of a single vesicle (reviewed in [1]). If for instance each vesicle is coupled to a single channel, then neurotransmitter release rate would be linearly proportional to the number of open channels, leading to a current cooperativity value of ~1, whereas this relationship would be non-linear if many channels contribute to a single exocytosis event, resulting in higher current cooperativity.

Due to the indirect nature of such current cooperativity assay, mathematical and computational modeling proved valuable in the analysis of this experimental protocol [2-7]. Here we use modeling of spatio-temporal calcium diffusion and buffering to explore more precisely the relationship between calcium current cooperativity and the underlying number of calcium channels contributing to vesicle release. We focus on the dependence of current cooperativity on the morphology of the active zone, in particular its dependence on the number of channels contributing to neurotransmitter release, both in the equidistant channel configuration, and for distinct non-equidistant channel distributions. Our general finding is that, under all conditions studied, current cooperativity significantly underestimates the number of channels contributing to the release of a single neurotransmitter vesicle, even when only a few channels contribute to release under condition of low release saturation. We also examine the implications of distinct current cooperativity values for the variability of synaptic transmission.
